# Fatigue crack growth of glass fiber laminates polymer composites using vacuum bagging technique

**DOI:** 10.1371/journal.pone.0345377

**Published:** 2026-04-15

**Authors:** Mariam Kadhiam Chaloob, Rafil Mahmood Laftah, Daniel Young, Tahseen A. Alwattar

**Affiliations:** 1 Department of Mechanical Engineering, College of Engineering, University of Basrah, Basrah, Iraq; 2 Department of Mechanical and Material Engineering, Wright State University, Dayton, Ohio, United States of America; Builders Engineering College, INDIA

## Abstract

Fatigue failure in composite polymer materials has attracted the attention of numerous researchers due to the extensive range of applications for these materials in aerospace, automotive, and biomedical industries. In this study, E-Glass fiber chopped strand mat was laminated with Epoxy LR625 using the vacuum bagging technique (VBT). We examined mode I fatigue crack growth related to translaminar failure in chopped strand mat-reinforced resin composite laminates through both experimental and numerical methods, utilizing the extended finite element method and direct cyclic approach. The main finding of this research is determining Paris coefficients of composite polymer material, fiberglass chopped strand mat/epoxy, fabricated by VBT with translaminar cracks. Paris law constants, C and m, were determined to be 4 × 10 ⁻ ¹² and 4.8584, respectively. The results obtained from both experimental and extended finite element method (XFEM) models of fatigue life are in good agreement, with a 6% error in predicted fatigue life. This research provides the determination of the Paris coefficients, which serve as a correction between fatigue and fracture mechanics for the composite materials. The growth of fatigue cracks can be characterized by the relationship between the crack propagation rate during each loading cycle and the fluctuations in the stress intensity factor (SIF).

## 1. Introduction

Fiber-reinforced, polymer matrix composite materials can exhibit high specific modulus and strength, and can be relatively economical compared with metals [1]. These properties make composite material preferred in many applications, such as glass fiber reinforced plastic (GFRP) blades for horizontal axis wind turbines (HAWT) [2,3]. However, failure due to fatigue crack growth poses a significant risk for components and structures subjected to cyclic loading, potentially compromising reliability and safety [4]. To address this issue, experimental characterization is required, but this is time consuming and expensive, and is often supplemented by computational methods such as finite element analysis (FEA) to validate experimental data and analyze more complex geometries. Extended Finite Element Method (XFEM) is often utilized to simulate crack behavior and its propagation [5].

Paris et. al. identified a classic correlation between fatigue and fracture mechanics [6,7], showing how fatigue crack growth can be characterized by the connection between the rate of crack propagation in each loading cycle and the variation of the stress intensity factor [7];


$$\frac{da}{dN}=C{\left(\Delta K\right)}^{m}$$ (1)

**Fig 1:**
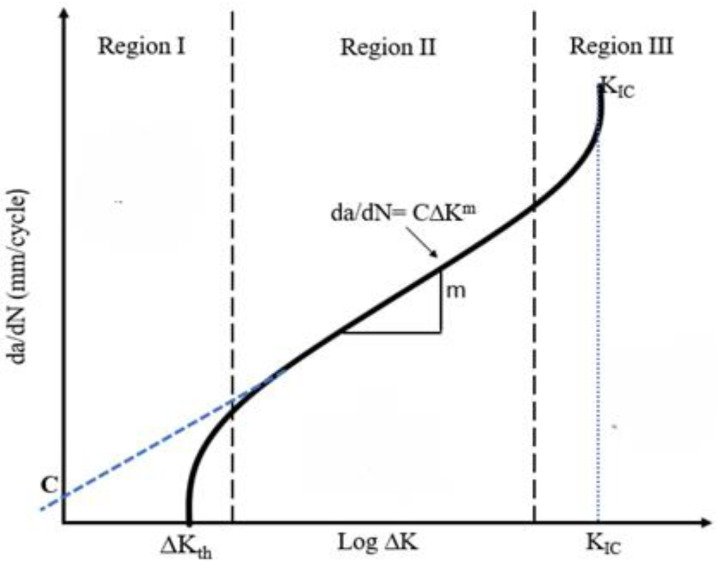
Crack growth rate vs. stress intensity factor range curve [6].

Where C and m are material constants obtained from experimental fatigue tests from fitting of the curve da/dN-ΔK as shown in Fig 1.

The (ΔK) represents the stress intensity factor range between maximum and minimum stress of cyclic fatigue load as:


$$\Delta K=f\Delta S\;\sqrt{\pi a}$$ (2)

where:


$$\Delta S=S_{max}-S_{min}$$ (3)

The $S_{max}$ and$\;S_{min}$ represents the maximum and minimum stress, respectively, f represents shape factor calculated in static loading conditions, and a refers to the crack length.

The shape factor f for mode I behavior [8,9] is equal to:


$$f\left(\frac{a}{b}\right)=\text{1}.\text{12}-\text{0}.\text{231}\left(\frac{a}{b}\right)+\text{10}.\text{55}{\left(\frac{a}{b}\right)}^{\text{2}}-\text{21}.\text{72}{\left(\frac{a}{b}\right)}^{\text{3}}+\text{30}.\text{39}{\left(\frac{a}{b}\right)}^{\text{4}}$$ (4)

Where b represents the width of sample.

The Paris equation estimates the number of cycles for the steady progression of a crack or defect from an initial detectable length to critical length. It is presumed that the crack propagates catastrophically once the critical length is attained. Conversely, crack growth does not occur when the stress intensity factor falls below a threshold value $K_{th}$ [10].

**Fig 2:**
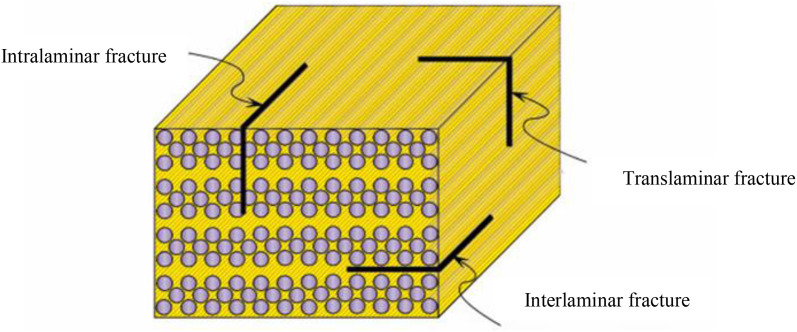
Fracture types in composite laminate [21].

Most reported studies focus on fatigue delamination growth (interlaminar fracture) using double cantilever beam (DCB), three-point end notched flexure samples (3ENF), and the single leg bending (SLB) method, for mode I, mode II, and mixed mode behavior, respectively, both experimentally and numerically. Computational approaches typically use a cohesive zone model (CZM) or virtual crack closure technique (VCCT) combined it with XFEM. Skvortsov et al. and Gur et. al. [11–14] studied delamination growth under mode I fatigue loading by CZM. Krueger et al. and Teimouri et al. [15,16] modelled delamination by VCCT under mixed mode and mode I, respectively. For mode I fatigue delamination, Tafazzolimoghaddam [17] presented a combination of CZM and XFEM, while Teimouri et al. [13] presented a combination of VCCT and XFEM. Experimentally, Al-Khudairi et al. [2] studied fatigue delamination under modes I and II. However, only limited studies, such as those by Bartaula et al. [18] and Gupta et al. [19], have employed the XFEM method solely for modeling fatigue crack growth, and these were conducted primarily in metals. The XFEM method was also applied to the study of functionally graded composite material plates by S. Bhattacharya et al. [20]. Fig 2 illustrates the differences between various fracture types observed in these materials [21].

The present study investigates fatigue crack growth due to translaminar fracture in composite laminate plates. The objective is to determine the Paris law constants experimentally and to simulate the behavior numerically using the XFEM with a direct cyclic approach under Mode I repeated loading, thus filling a gap in the current literature.

The XFEM method is a numerical approach that expands on the conventional finite element method (FEM), allowing the solution of differential equations with discontinuous functions. XFEM can addressing computational challenges associated with localized features that cannot be adequately resolved by mesh refinement [22]. One of the first applications of this methodology was modelling cracking in materials [22]. In this preliminary formulation, crack opening displacement was accounted for using bases that include discontinuous functions, which were implemented where the crack intersects the element, in contrast to standard polynomial bases for finite elements. One major advantage of XFEM over conventional mesh update techniques is that the mesh can remain static and does not need to be updated at every time step to track the evolution of the crack [23]. The method also applies to a wider range of problems in the presence of materials interfaces and singularities, in the uniform meshing of microstructural lineaments such as voids, or any other problem where a proper set of basis functions can be found to develop localized features [23]. This can significantly improve accuracy and convergence rates in some cases. Moreover, XFEM, which allows the elimination of remeshing of discontinuity surfaces, where the discontinuity is localized along the edges of the mesh, leads to reduced computational costs and a possible reduction in projection errors [23]. The notion of XFEM is based on the concept of domain enrichment. As a result, it is capable of accurately representing some features of these discontinuities related to cracks and interface areas. XFEM can be viewed as a local version of the PUFEM enrichment in a local domain; it ultimately relies on the development of an external enrichment for crack modelling, using meshless approaches such as EFG, and Hp-clouds [23]. Early approximations of XFEM [23] utilized the enrichment approximation function u(x) illustrated in equation (5).


$$u^{h}(x)=u^{FE}+u^{enr}=\sum_{j=\text{1}}^{n}N_{j}(x)u_{j}+\sum_{k=\text{1}}^{m}N_{k}(x)\psi (x)a_{k}$$ (5)

$N_{j}(x)$ refers to the usual nodal shape functions, $u_{j}$ is the vector of uniform degrees of nodal freedom in the FEM, $a_{k}$ is the additional set of freedom degrees to the conventional FE model, and ψ(x) is the discontinuous enrichment function [23,24].

## 2. Materials and methods

**Fig 3:**
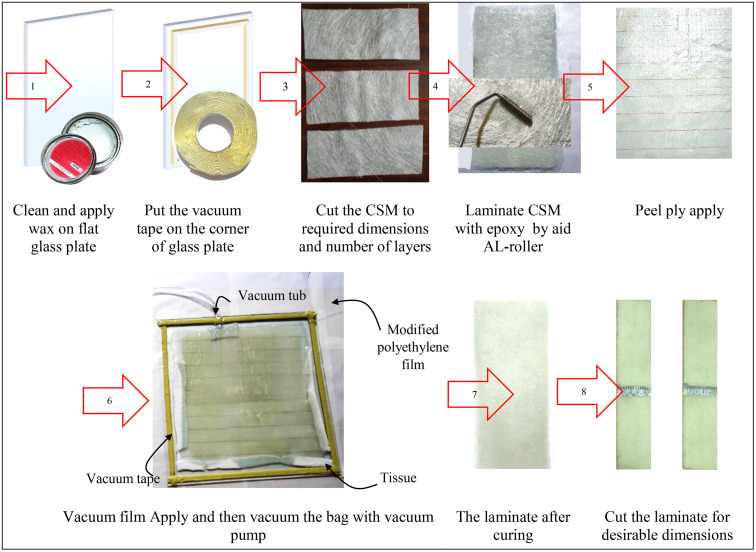
Vacuum bagging process.

We utilized the vacuum bagging technique (VBT), which offers several advantages for composite laminates, including reduced void content [25], enhanced fiber containment [26], improved fiber wet-out through applied pressure, and minimized volatile emissions during curing [27]. E-glass fiber Chopped Strand Mat (CSM) was used as fiber reinforcement, conforming to specification EMCL450–1250 [28], laminated with LR625 epoxy resin and hardener using the VBT. The vacuum bagging process is illustrated in Fig 3. Initially, the glass plate is thoroughly cleaned, and a layer of wax is applied to its surface to prevent adhesion between the composite laminate and the plate. Double-sided vacuum tape is then affixed along the edges of the glass plate using its bottom adhesive face. Three layers of chopped strand mat (CSM) are cut to appropriate dimensions to fabricate three test samples. Each ply has a nominal thickness of 0.7 mm, resulting in a total laminate thickness of approximately 2 mm upon completion. The CSM layers are impregnated with epoxy resin using a conventional hand lay-up method. A layer of nylon peel-ply (release fabric) is subsequently applied to the final layer to ensure a smooth surface finish. An absorbent tissue layer is then placed to remove any excess resin. The lay-up is covered with a modified polyethylene film, which is sealed using the top adhesive face of the vacuum tape, while accommodating the placement of the vacuum tube. The assembly is then subjected to vacuum pressure, and curing is performed at ambient temperature for 24 hours. Upon completion of the curing process, the composite laminate is trimmed to the specified dimensions.

### 2.1. Tensile testing

**Fig 4:**
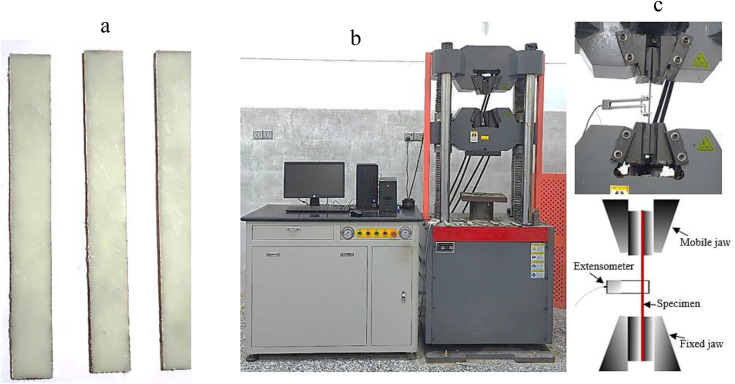
Tensile Testing Setup and Specimens Fabricated via VBT (ASTM D3039). (a) three tensile test specimens created using VBT (ASTM D3039), (b) tensile test machine, and (c) specimen fixed in jaws with extensometer.

To validate the mechanical properties of the CSM/epoxy composite, three specimens were subjected to tensile testing, as shown in Fig 4, and prepared in accordance with ASTM D3039 [29]. The results demonstrated a high degree of sample-to-sample consistency, as presented in Tables 1 and 2.

**Table 1:** Mechanical properties of CSM/Epoxy composite laminates.

CSM/Epoxy composite	Tensile strength (MPa)	Tensile Modulus (GPa)	Tensile Strain %
Sample 1	157.05	6.73	4.52
Sample 2	176.21	8.64	2.08
Sample 3	151.51	8.18	1.60
Sample 4	159.23	7.12	3.45
Sample 5	165.01	8.23	2.90
average	161.80 ± 3.75	7.78 ± 0.32	2.91 ± 0.45

**Table 2:** Validation of CSM/Epoxy tensile test results through comparison with previous studies.

References	Current study	K. Bijesh [30]	Ravikumar&Prasad [31]
Material	CSM/Epoxy	CSM/Epoxy	CSM/Epoxy
Composite manufacturing	Vacuum bagging	hand lay-up technique	hand lay-up technique
V_*f*_%	60	50	60
Tensile modulus (GPa)	7.78	6.23	7.52

Additionally, Poisson’s ratio was determined to be 0.3, and the fracture energy was calculated as 43.29 J/mm^2^.

### 2.2. Fatigue testing

**Fig 5:**
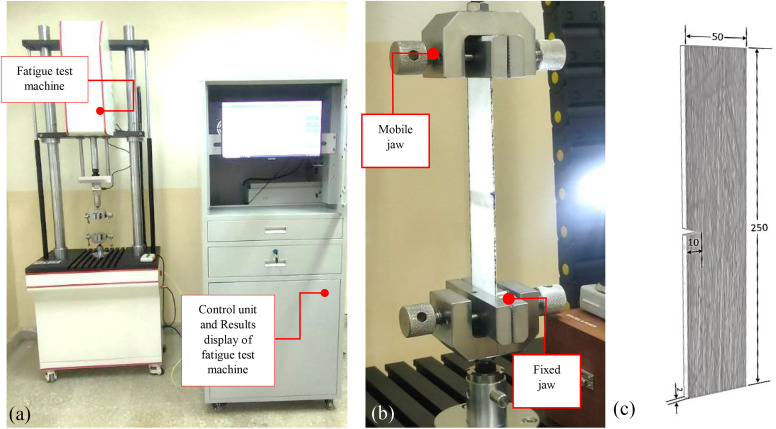
Fatigue Testing Setup and Specimen Configuration. (a) LR-QBPL-5000N fatigue test machine and unit control, (b) fixing the specimen in jaws, and (c) dimensions of specimen in mm.

**Fig 6:**
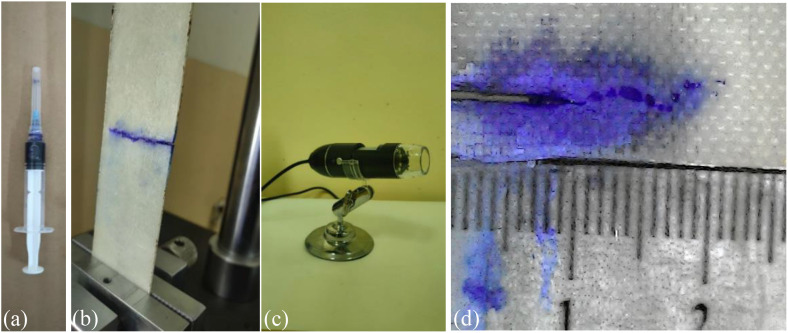
Tools for detecting crack length: (a) ink needle (b) illustrated of crack growth path by ink (c) digital microscope camera (d) image by digital microscope camera.

Fatigue testing was conducted to obtain data on the relationship between crack length and the number of loading cycles. This data was used to calculate the crack growth rate (da/dN), which was subsequently plotted against the stress intensity factor range (${\Delta 𝐊}_{𝐈}$) to derive the Paris curve. Fatigue testing was conducted on five CSM/epoxy composite samples, with dimensions shown in Fig 5, in accordance with ASTM E647-15 [32]. Each specimen was subjected to cyclic loading at different stress levels (24, 29, 34, 39, and 44 MPa) using a frequency of 5 Hz, employing a LR-QBPL-5000N fatigue testing machine, as illustrated in Fig 5. Crack propagation was monitored using a combination of ink marking, ruler paper, and a digital microscope camera, as shown in Fig 6.

## 3. Results and discussion

### 3.1. Fatigue testing results

**Fig 7:**
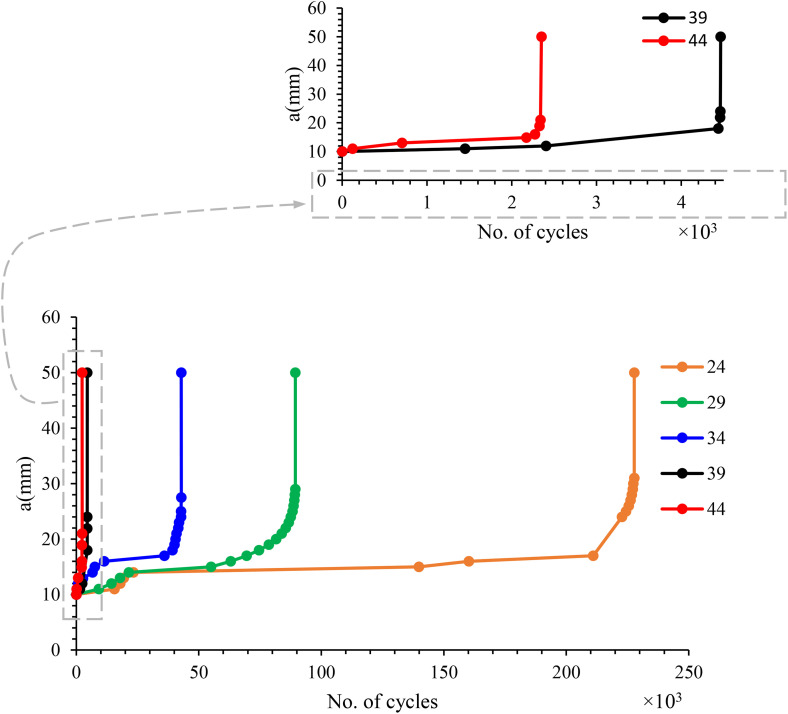
Crack growth length vs number of cycles under different repeated stress levels in MPa & initial notch (a_i_)=  10 mm.

Fatigue testing was conducted on five CSM/epoxy composite specimens in accordance with ASTM E647-15. The crack length was recorded as a function of the number of loading cycles, as shown in Fig 7, which illustrates crack propagation behavior under distinct stress levels: 24, 29, 34, 39, and 44 MPa, with an initial notch (a_i_) of 10 mm. A rapid increase in crack length was observed during both the initial and final stages of cyclic loading. Between these phases, a plateau region representing stable crack propagation was evident, particularly at the 24, 29, and 34 MPa stress levels. This behavior is attributed to the material’s ability to redistribute load and dissipate energy at these stress levels. In contrast, at higher stress levels (39 and 44 MPa), catastrophic failure occurred more quickly, with a significantly smaller stable crack growth region. These results indicate that fatigue life is inversely proportional to the applied stress level.

**Fig 8:**
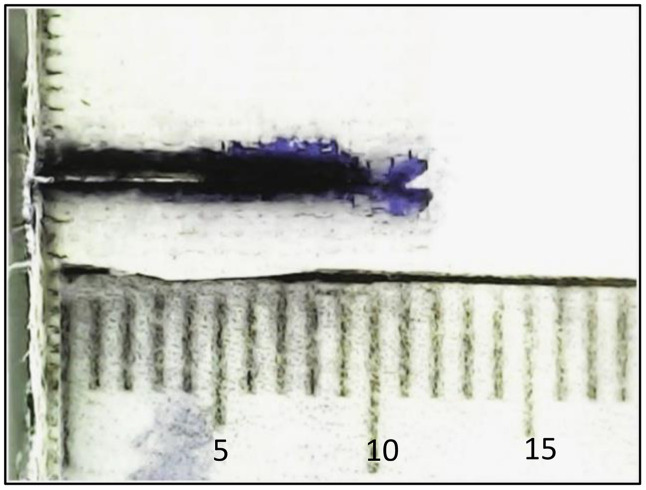
Beginning of crack growth at N = 2409 cycles.

The specimen subjected to fatigue loads of 24, 29, and 34 MPa exhibited notably longer fatigue life. This extended fatigue life is attributed not only to the lower applied stress but also to the occurrence of crack branching during the early stages of cyclic loading, as illustrated in the crack image in Fig 8. The reduced energy available for crack propagation at these load levels likely contributed to the branching behavior, where some cracks were arrested while others continued to propagate, depending on the local material microstructure.

**Fig 9:**
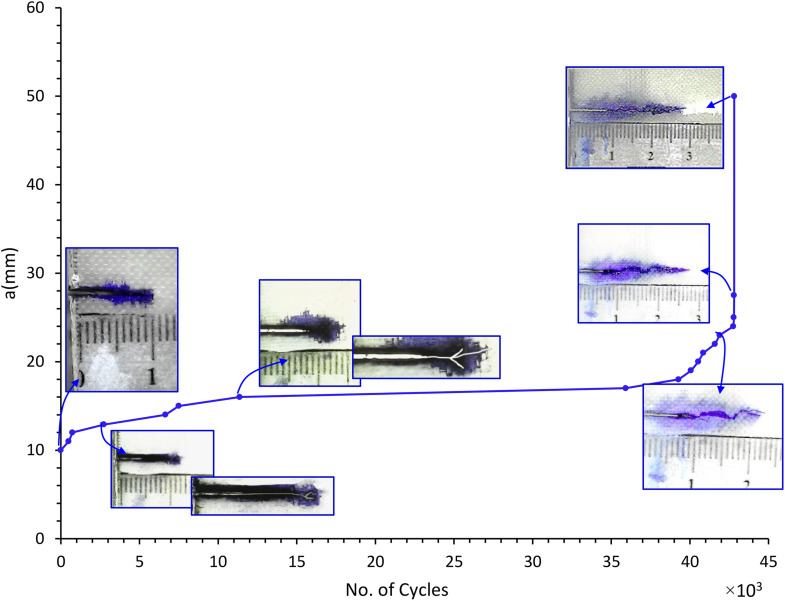
Crack growth behavior of CSM/Epoxy under repeated stress level (34 MPa).

Fig 9 illustrates the crack growth behavior over the loading cycles for the specimen tested at 34 MPa, showing a stable crack growth phase (plateau region) occurring between 5,000 and

**Fig 10:**
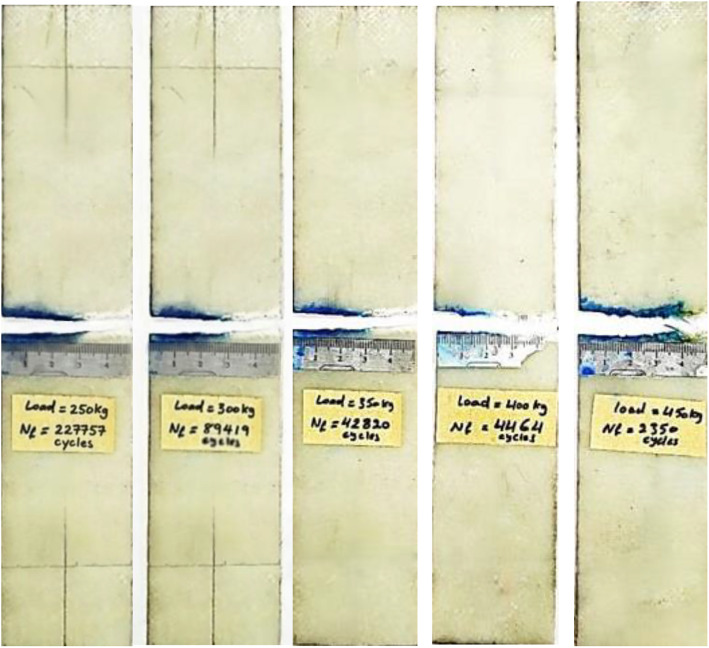
Fatigue fracture behavior of three CSM/epoxy composite specimens.

35,000 cycles, during which the material effectively absorbs energy and redistributes the load within the composite. Crack propagation likely results primarily from fiber-matrix debonding and matrix cracking. The blue region in the figure denotes areas of damage associated with these mechanisms. Unlike metals, the crack behavior in the composite does not exhibit a distinct crack tip. Between 35,000 and 43,000 cycles, rapid crack propagation occurs, with the crack length increasing exponentially until catastrophic failure. The final image highlights fiber breakage and pull-out, represented by the white region. The catastrophic failure of the three specimens started around the specimen half-width where the stress intensity factor equals fracture toughness. This behavior must be taken into account during engineering design. The same behavior was observed for the specimens tested at 24 and 29 MPa, with stable crack growth occurring between 23,000 and 211,000 cycles and between 21,000 and 63,000 cycles, respectively. Rapid crack growth occurred during the remaining cycles until final failure. Fig 10 shows the final failure for all five specimens.

**Fig 11:**
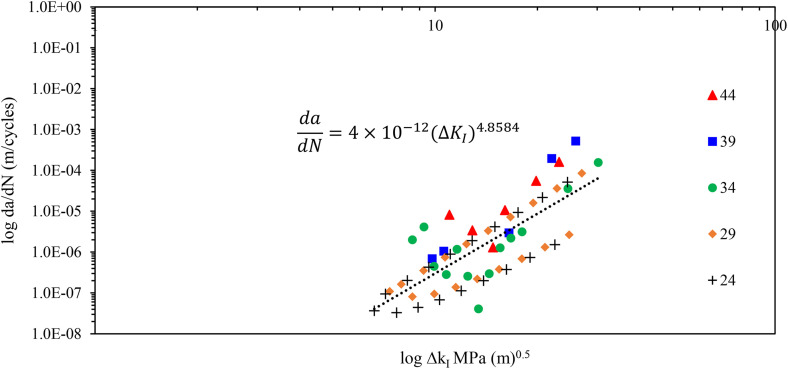
Crack growth rate vs stress intensity factor range of CSM/Epoxy composite laminate.

Data on crack length vs number of cycle curves was collected to determine the crack growth rate and stress intensity factor range, where the shape factor was calculated according to equation 4. Fig 11 shows the Paris curve. The slope of the line represents the Paris exponent, defining the sensitivity of crack growth rate to ΔK_I_, while the intercept represents the Paris coefficient. Stress levels of 39 and 44 MPa exhibit accelerated crack propagation rates, as indicated by the higher positioning of square and triangle markers. In contrast, the 24, 29 and 34 MPa stress levels exhibit reduced crack growth rates. The observed scatter in the data points reflect the inherent variability in microstructural damage mechanisms, including fiber-matrix debonding and matrix cracking, characteristic of composite materials. Additionally, crack closure effects at lower ΔK values may contribute to deviations in the measured data. The Paris law constants, C and m, were determined to be 4 × 10 ⁻ ¹² ± 1.26 × 10^-13^and 4.85 ± 0.51, respectively.

Table 3 summarizes previous studies on glass fiber–reinforced composites and shows how the Paris-law coefficient and exponent are affected by several factors, including matrix type, manufacturing process, fiber nature (long or short), notch geometry, and fiber content expressed as weight percent (wt.%) and volume percent (vol.%).

**Table 3:** Research of Paris law relation with stress intensity factor and strain energy release rate.

Specimen & Material	Relation	Relation parameter value		Ref.
		Paris Law coefficient m/cycle	Paris Law exponent	
CT specimen of PP+short glass fiber (SGF) samples with wt. % (10–40)-vol. % (3.9–19.4)	$\frac{da}{dN}=C(\Delta k{)}^{m}$	Notch L		[33]
		5.5 × 10^-8^-3.7 × 10^−10^	7.8-9	
		Notch T		
		3 × 10^-8^-2.88 × 10^-13^	8.7-11.5	
CT specimen of PP + long glass fiber (LGF) samples with wt. % (10–40)-vol. % (3.9–19.4)	$\frac{da}{dN}=C(\Delta k{)}^{m}$	Notch L		
		3.8 × 10^-7^-9 × 10^-13^	5.3-10.1	
		Notch T		
		1 × 10^-9^-5.9 × 10^-14^	7.8-13	
CT specimen of Sheet molding compound (SMC) of chopped E-glass/polyester	$\frac{dA}{dN}=C{\left({\Delta k}^{\alpha }\cdot {k_{ave}}^{\beta }\right)}^{m}$	2.06 × 10^-20^	11.2	[34]

### 3.2. XFEM simulations

Fatigue simulation was conducted using the XFEM method and the direct cyclic approach. In this study, a quasi-static analysis framework is adopted, integrating time-domain techniques with a truncated Fourier series representation to iteratively capture the stabilized cyclic response of structures, which is applicable to materials that exhibit nonlinear material behavior. By circumventing the computational demands typically associated with full transient simulations, it offers significant efficiency advantages, particularly for large-scale systems subjected to numerous load cycles. The method applies to both linear materials and nonlinear materials undergoing localized plastic deformation. The direct cyclic approach simulates fatigue crack growth based on the Paris law, but expressed in terms of energy release rate rather than the stress intensity factor of (eq.1), as shown in the following equation:


$$\frac{da}{dN}=C_{\text{3}}{\Delta G}^{C_{\text{4}}}$$ (6)

Where:


$$C_{\text{4}}=\frac{m}{\text{2}}$$ (7)

$$C_{\text{3}}=C{\left(E^{\prime }\right)}^{C_{\text{4}}}$$ (8)

The $E^{\prime }=E$ for plane stress and $\;E^{\prime }=E/\text{1}-{\;v}^{\text{2}}$ for plane strain, where 𝐸 is the elasticity Modulus, and 𝜈 is Poisson’s ratio [35].

The energy release rate, G, was calculated using the following relation:


$$G=\frac{\overset{\text{2}}{\underset{I}{K}}}{E^{\prime }}$$ (9)

where $K_{I}$ is the mode I stress intensity factor, and E′ denotes the effective modulus accounting for plane stress conditions. Numerical simulation was carried out using ABAQUS 2017, incorporating the calculated energy release rate and the required Paris law constants for fatigue crack propagation modeling. The simulation was applied to the specimen geometry shown in Fig 5, subjected to a cyclic fatigue load with a peak stress amplitude of 44 MPa. This approach allowed for the accurate estimation of fatigue crack growth behavior under translaminar fracture conditions in the composite laminate.

**Fig 12:**
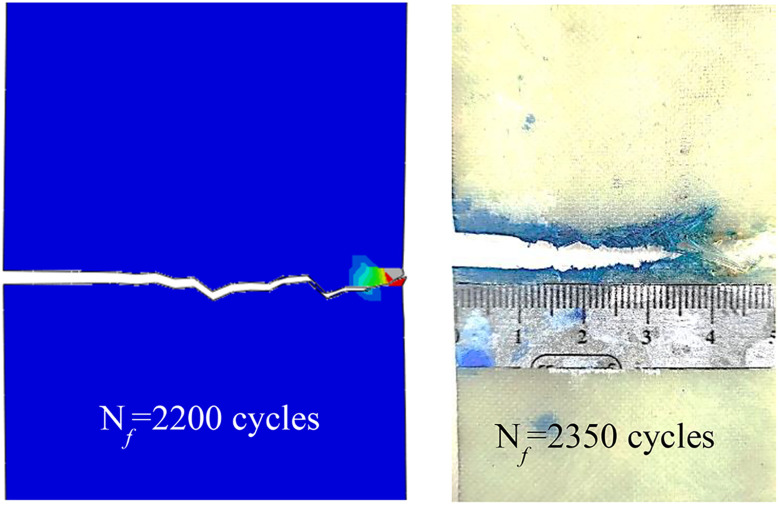
Numerical and experimental fatigue test results of specimen with 44 MPa repeated stress level.

The results indicate a discrepancy of approximately 6% between the fatigue life predicted by the numerical simulation and the experimentally observed values. Specifically, the simulated fatigue life was 2200 cycles, whereas the experimental fatigue life was measured at 2350 cycles. Fig 12 presents a comparison between the simulation results and the experimental data.

**Fig 13:**
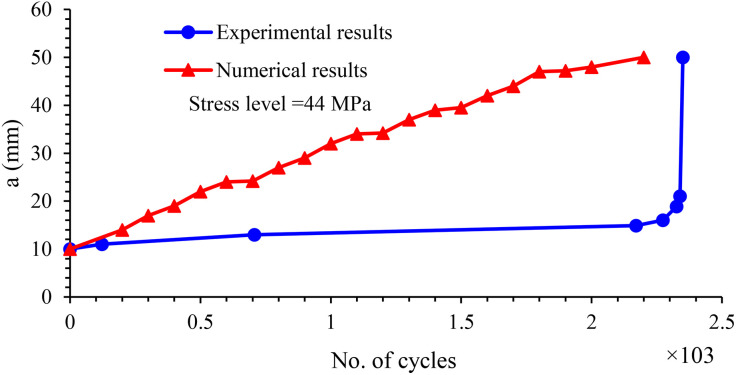
Comparison between crack growth behavior for experimental and numerical results.

The crack length against the number of cycles was plotted for experimental and numerical ones, as in Fig 13.

The numerical simulation exhibits a relatively monotonic increase in crack length with respect to cycle count. This trend results from the use of the direct cyclic approach in ABAQUS, where the Paris law is uniformly applied along the entire width of the specimen. While this method is effective for predicting the total fatigue life, it does not capture the progressive nature of crack growth observed in real materials. Specifically, ABAQUS lacks the ability to model the catastrophic crack propagation phase, which typically occurs near failure. As a result, the simulation assumes that the entire crack path undergoes stable crack growth, governed by the Paris law.

**Fig 14:**
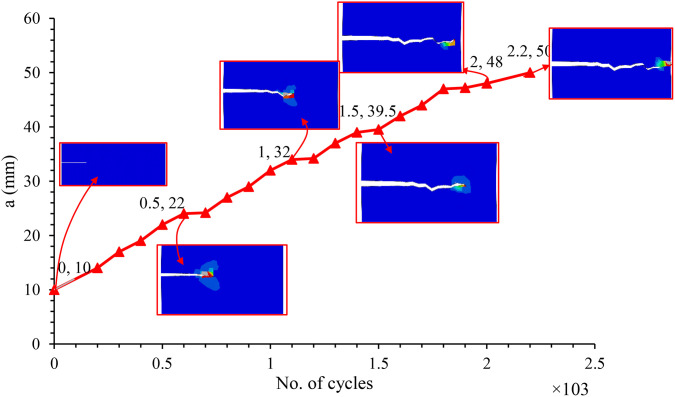
Crack growth behavior during repeated stress level (44 MPa) in numerical simulation of CSM/Epoxy.

**Fig 15:**
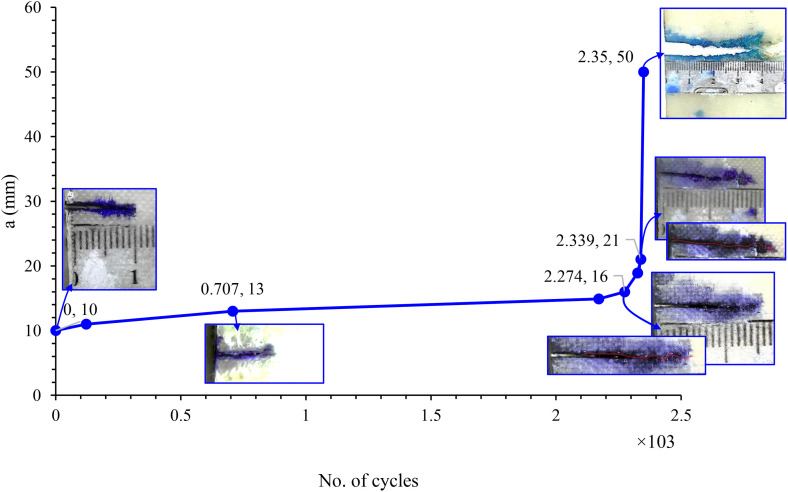
Crack growth behavior during repeated stress level (44 MPa) in experimental test of CSM/Epoxy.

In contrast, the experimental data show that the stable crack growth region is localized—extending from the initial notch to approximately the mid-width of the sample—beyond which crack growth acceleration occurs, ultimately leading to catastrophic failure. Therefore, while the Paris law is applicable in the experimental work only within the stable growth region, the numerical model overestimates this region by applying it across the full width of the specimen. This discrepancy is evident in Figs 14 and 15, which illustrate the differences in crack propagation behavior between the numerical and experimental results. The simulation yields a smooth, gradual crack extension profile, whereas the experimental data demonstrate more complex behavior with a clear transition from stable to unstable crack growth.

The experimental results for crack growth under cyclic loading exhibit a non-linear progression, primarily influenced by the heterogeneous nature of the composite material. In contrast, the numerical simulation results show a more linear crack growth behavior, as they are based on the assumption of material homogeneity and do not account for microstructural defects such as voids or resin-rich regions. This simplification contributes to a faster predicted crack propagation rate, as represented by the red curve. Additionally, during experimental testing, fatigue loading may induce load relaxation and non-uniform stress distribution, further affecting crack development. In comparison, the numerical simulation assumes idealized cyclic loading conditions, which may lead to a slight underestimation of the actual fatigue life.

## 4. Optical analysis

**Fig 16:**
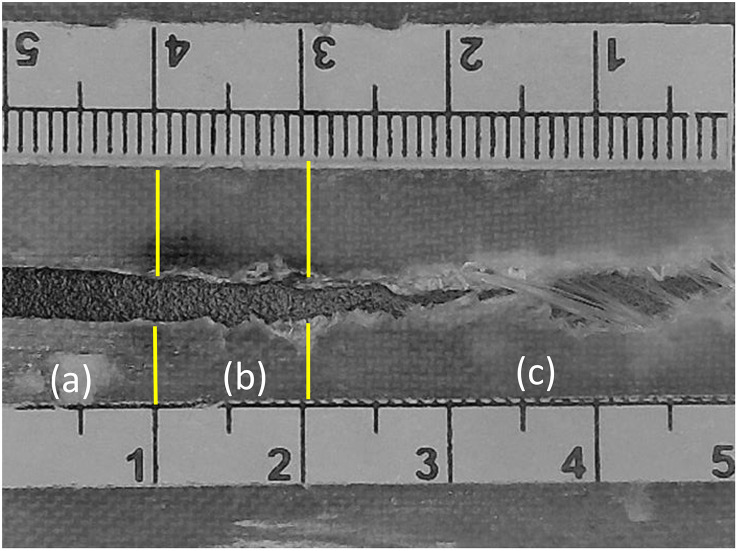
Optical analysis for fatigue sample (44 MPa). (a) Initial notch region – In this zone, both the fibers and the matrix are completely cut across approximately 10 mm of the specimen width, corresponding to the machined notch as illustrated in Fig 17 (a1). (b) Stable crack growth region – In this region, the crack advances gradually under cyclic loading. Fiber breakage occurs along the crack front and is accompanied by matrix cracking, with only limited fiber pull-out observed, as shown in Fig 17 (b1&b2). The length of this stable region increases as the applied fatigue stress level decreases, indicating that lower loads promote a longer period of controlled crack growth before final failure. (c) Catastrophic crack growth region – In this final region, the crack accelerates rapidly and propagates through most of the remaining specimen width. Extensive fiber pull-out, fiber–matrix debonding, and localized crazing are visible as illustrated in Fig 17 (c1). The extent of this catastrophic zone decreases when the applied fatigue load is reduced, which is consistent with lower crack growth rates at lower stress levels.

**Fig 17:**
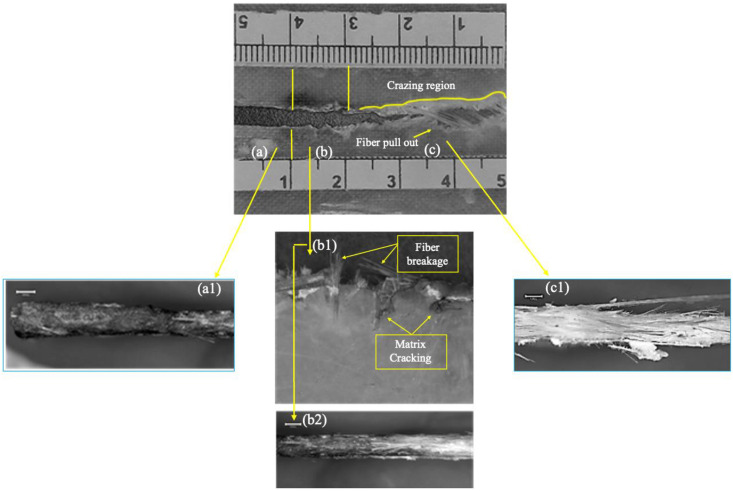
(a1) Initial notch region, (b1&b2) Stable crack growth region, and (c1) Catastrophic crack growth region. Region C (catastrophic crack growth region) is observed along the tensile test sample, resulting from the increased stress over time during the test, which ultimately leads to fracture, as shown in Fig 18. This region exhibits more extensive crazing compared to the fatigue test sample, as depicted in Fig 18b. This difference highlights the impact of the applied load on the sample’s failure behavior.

Optical analysis was carried out using a BestScope-BMM-4K8MPA digital microscope at 20 × magnification to examine the fatigue fracture surface of the specimen tested under a maximum stress of 44 MPa, as shown in Fig 16. The crack path can be divided into three characteristic regions: (a) the initial notch region, (b) the stable crack growth region, and (c) the catastrophic crack growth region Fig 17.

**Fig 18:**
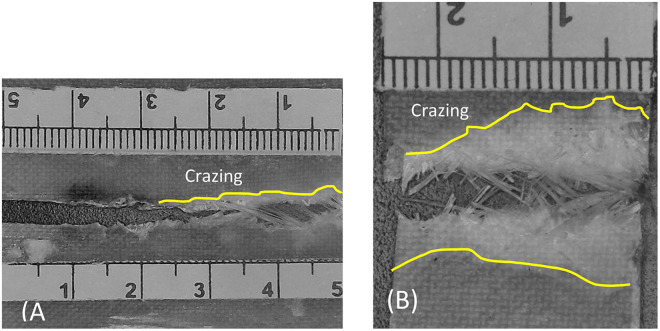
Comparison between (a) fatigue sample and (b) tensile sample.

Fig 18 compares the fracture appearance of the fatigue-tested specimen with that of a specimen failed in monotonic tension. The fatigue fracture surface (Fig 18a) clearly exhibits the three regions described above, whereas the tensile fracture surface (Fig 18b) shows a more uniform failure. This comparison highlights the strong influence of cyclic loading on the development of damage and crack growth mechanisms in the CSM/epoxy laminates.

## 5. Conclusion

The adoption of composite materials in advanced applications inevitably exposes them to fatigue loading. Accordingly, this study conducted fatigue testing on CSM/epoxy composite laminates, guided by principles of linear elastic fracture mechanics (LEFM). Simulations using the direct cyclic approach and XFEM revealed that catastrophic crack initiation occurs near the half-width of the specimen, an essential design consideration. Furthermore, early-stage crack branching during cyclic loading significantly extended fatigue life, a phenomenon that corroborates reports in fatigue fracture of composites and metals where micro-scale bifurcations introduce retardation effects and increase resistance to crack growth. The direct cyclic simulation showed strong agreement with experimental fatigue life data, yielding only a 6% discrepancy, despite its limitations in accurately reproducing crack growth behavior. This pattern aligns with findings by Hofman et al. [36], who successfully applied XFEM-based fatigue modeling to composite laminates, demonstrating accurate fatigue life predictions but highlighting challenges in capturing complex damage evolutions. Given the high computational cost and resource intensity of fatigue testing, the strong numerical–experimental agreement supports the use of simulation as a viable alternative for fatigue-life estimation, especially in the preliminary design phase. However, the observed divergence in crack propagation paths underscores the need for more advanced modeling techniques that incorporate microstructural heterogeneity, such as cohesive-zone models or phase-field methods. These methods offer improved fidelity in simulating damage mechanisms like fiber-matrix debonding, void formation, and crack bifurcation. In addition, optical analysis of the fatigue-fractured specimens provided direct microstructural evidence for the crack growth mechanisms inferred from the Paris-law and XFEM results. The observed subdivision of the crack path into an initial notch region, a stable crack growth region, and a catastrophic failure region, together with the transition from predominantly matrix cracking and fiber breakage to extensive fiber pull-out and crazing at higher ΔK, confirms the strong interaction between local damage modes and global crack growth rate. These observations not only validate the macroscopic fatigue crack growth trends reported in this study but also highlight the critical role of fiber–matrix interactions and damage localization in controlling the fatigue performance of CSM/epoxy laminates.

## Supporting information

S1 File Supporting information data. (ZIP)
